# Effects of aromatherapy on sleep quality in patients: Protocol for an umbrella review

**DOI:** 10.1371/journal.pone.0329928

**Published:** 2025-08-18

**Authors:** Hongrui Shi, Mengqi Liu, Xinxin Fan, Wei Peng, Yan Hu

**Affiliations:** 1 College of Nursing, Shanxi University of Chinese Medicine, Jinzhong, China; 2 Department of Urology/Pelvic Floor and Andrology, West China School of Public Health and West China Fourth Hospital, Sichuan University, Chengdu, China; 3 Department of Oncology, West China School of Public Health and West China Fourth Hospital, Sichuan University, Chengdu, China; 4 Research Center for Palliative Care, West China-PUMC C.C. Chen Institute of Health, Sichuan University, Chengdu, China; Babol University of Medical Sciences, IRAN, ISLAMIC REPUBLIC OF

## Abstract

**Introduction:**

Poor sleep quality affects disease recovery and overall quality of life. Aromatherapy has been shown to improve sleep quality by alleviating anxiety, promoting relaxation, and enhancing blood circulation. Several meta-analyses have reported the effects of aromatherapy on sleep quality, however, the efficacy varies widely, with some studies showing that the effects are negligible or inconsistent. A comprehensive review synthesizing the available evidence on the impact of aromatherapy on sleep quality is still needed. Therefore, this study aims to provide an umbrella review summarizing all available systematic reviews and meta-analyses investigating the effects of aromatherapy on sleep quality.

**Methods and analysis:**

A thorough literature search will be conducted across six English-language databases and four Chinese-language databases, following the Joanna Briggs Institute (JBI) methodology for umbrella reviews. Two reviewers will independently screen all articles to determine their eligibility based on pre-established inclusion criteria. Additionally, a manual search will be performed by reviewing the reference lists of the included studies. Data extraction will be conducted from all eligible studies, and the quality will be assessed using the Assessment of Multiple Systematic Reviews-2 (AMSTAR 2) tool. The study selection process will be presented using a PRISMA flow diagram. Statistical analyses will be performed using appropriate statistical methods to summarize and describe the findings.

**Results:**

This study aims to provide comprehensive evidence by systematically evaluating the effects of aromatherapy on sleep quality in patients. It seeks to uncover the impact of aromatherapy on sleep quality across different patient populations and explore variations in its effectiveness based on disease type and the form of aromatherapy used. We will conduct a systematic evaluation of all eligible systematic reviews and meta-analyses. Subgroup analysis will also be performed, if applicable, to divide patients into groups according to how aromatherapy attributes (e.g., population type, dosage, route of administration) and disease types affect the effect size.

**PROSPERO registration number:**

CRD42024580250.

## 1. Introduction

Sleep, a cornerstone of health and recovery from illness, is frequently disrupted and insufficient among patients, placing them at an increased risk for sleep disorders. Sleep disorders are persistent conditions characterized by difficulties in sleep initiation, maintenance, or abnormal sleep patterns [1]. According to the International Classification of Sleep Disorders (ICSD-3) [2], sleep disorders primarily include insomnia disorders, sleep-related breathing disorders, central disorders of hypersomnolence, and other sleep-related conditions. The literature highlights a high prevalence of sleep disorders across various patient populations, with rates reported as high as 80% in cancer patients [3], 94.3% in end-stage renal disease (ESRD) or chronic kidney disease (CKD) patients undergoing hemodialysis [4], and nearly 90% among the elderly [5]. Critically ill patients also exhibit a substantial incidence (66%) [6], largely attributable to environmental factors such as noise, light, and nursing activities [7]. Poor sleep quality hinders recovery and survival, impairs cognitive function, prolongs treatment duration, and increases the risk of comorbidities such as diabetes and cardiovascular diseases [8,9]. Therefore, implementing effective strategies to improve sleep quality in patients is crucial.

Pharmacotherapy, a common intervention for sleep disorders, can effectively relieve symptoms but is associated with side effects such as cognitive impairment and addiction [10]. Furthermore, long-term use of hypnotic medications may lead to a decline in sleep quality [11]. Compared to conventional treatments, Complementary and Alternative Therapies (CAT) provide cost-effective and versatile advantages, with evidence supporting their efficacy in improving sleep quality. Aromatherapy, a widely recognized CAT modality, employs essential oils and aromatic plant extracts to influence emotional and physiological states. It has been empirically demonstrated to alleviate anxiety, promote relaxation, enhance circulation, and ultimately improve sleep quality [12,13].

Researches have indicated that aromatherapy may offer therapeutic benefits for sleep disturbances, but efficacy varies. Several systematic reviews and meta-analyses have reported the effects of aromatherapy on sleep quality. Although these reviews provide valuable insights into specific aromatherapy applications, such as inhalation and massage, they are constrained by various factors: limitations in study design, inconsistencies in intervention assessment techniques, and variations in outcomes, thereby complicating the derivation of definitive conclusions. A comprehensive review of the evidence regarding aromatherapy’s impact on sleep quality is still lacking. To enhance the effectiveness of aromatherapy in improving sleep quality in patients, it is essential to systematically evaluate the methodological quality, potential biases, and validity of all available studies on this topic.

This study aims to provide comprehensive evidence by systematically evaluating the effects of aromatherapy on sleep quality in patients. It seeks to uncover the impact of aromatherapy on sleep quality across different patient populations and explore variations in its effectiveness based on disease type and the form of aromatherapy used. Furthermore, the study intends to identify the most effective treatments for improving sleep quality in patients. To achieve these objectives, we will conduct a systematic evaluation of all eligible systematic reviews and meta-analyses. Specifically, the study will address two key questions: (1) Is aromatherapy effective in improving sleep quality in patients? (2) If so, are the effects influenced by specific factors such as the type of disease, the form of aromatherapy, the duration of the intervention, or other variables?

## 2. Methods and analysis

### 2.1. Registration

This study will adhere to the Joanna Briggs Institute (JBI) methodology for umbrella reviews [14] and follow the Preferred Reporting Items for Systematic Review and Meta-Analysis Protocols (PRISMA-P) guidelines [15] (S1 File). The protocol was officially registered in PROSPERO on August 26, 2024. (Registration number: CRD42024580250).

### 2.2. Inclusion criteria

#### 2.2.1. Types of studies.

This umbrella review will include Systematic reviews, with or without Meta-analysis, that incorporate randomized controlled trials (RCT) or observational studies. Case reports, original studies that are not part of systematic reviews, research protocols, clinical guidelines and letters will be excluded. Additionally, literature where data cannot be extracted or transformed will not be included.

#### 2.2.2. Types of participants.

The review will focus on adult patients (aged 18 or older) without restrictions on country’s income level (low, middle, or high), the settings (community, hospital, clinic, institution), and disease type. Included diseases may encompass both acute and chronic conditions (e.g., cancer, critical illness, hemodialysis, post-surgery, etc.).

#### 2.2.3. Types of interventions.

Any type of aromatherapy (e.g., lavender essential oil, rose essential oil, peppermint essential oil, chamomile essential oil or compound essential oils) will be considered, regardless of dosage or route of administration. Route of administration may include massage, inhalation, and other methods.

#### 2.2.4. Comparisons/Control.

This review will evaluate systematic reviews of studies that may or may not include a control group. Control groups can consist of blank controls, wait-lists, placebos, usual care, or standard treatments.

#### 2.2.5. Outcomes.

Only studies that assess sleep quality as primary outcome will be included. Sleep quality in the umbrella review will be measured using the Pittsburgh Sleep Quality Index (PSQI) [16] or other standardized scales. The PSQI evaluates subjective sleep experiences over the past month, with total scores ranging from 0 to 21, where higher scores indicate worse sleep quality.

### 2.3. Search strategy

A comprehensive literature search will be conducted across six English and four Chinese databases: PubMed, Cochrane Library, JBI database, Web of Science, Embase, Cumulative Index to Nursing and Allied Health Literature (CINAHL), Chinese National Knowledge Infrastructure (CNKI), Wanfang database, Chinese science and Technology Journal Database (VIP), and Chinese Biomedical Literature Database (CBM) from inception to April 2025. Manual searches will be performed by reviewing the reference lists of included studies. Studies in both Chinese and English will be included. The search will utilize both MeSH terms and text words. The detailed search strategy and initially organized search terms for each database are presented in S2 File.

### 2.4. Study selection

**Fig 1:**
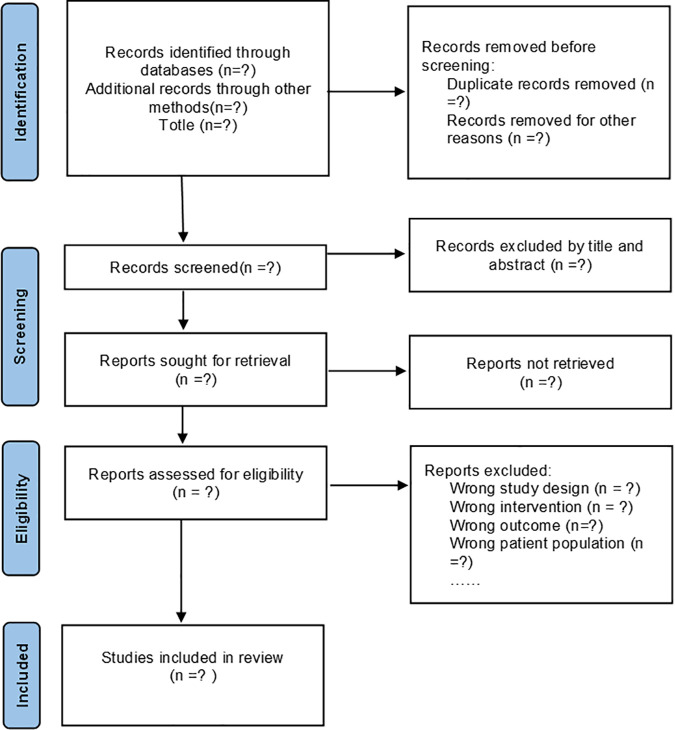
The PRISMA flow diagram of the study selection process.

All references extracted from the ten databases will be imported into Zotero 7, and duplicates will be removed. Two reviewers will independently screen titles and abstracts to identify studies that potentially meet the inclusion criteria. Studies that clearly do not meet the inclusion criteria will be excluded at this stage. The full texts of remaining studies will be examined during secondary screening. Any disagreements between two reviewers will be resolved by consensus. If consensus cannot be reached, a third reviewer will be consulted to make the final decision. Reasons for exclusion will be documented. All stages of the selection process will adhere to the guidelines presented in the PRISMA flow chart [17]. The literature screening process is illustrated in Fig 1.

### 2.5. Data extraction

Data will be recorded in a standard data extraction form. Two reviewers will independently extract data from eligible studies, including study characteristics (publication year, countries of origin, study design, number of original studies, and inclusion/exclusion criteria), participant characteristics (sample size, age, gender, and type of diseases), interventions and comparisons (dosage and route of administration), methodology aspects (quality assessment, data extraction process, and heterogeneity assessment), outcomes, adverse effects, and conclusions. Missing data will be retrieved by contacting the original author via email. Discrepancies will be resolved by consensus, and if necessary, a third reviewer will be consulted.

### 2.6. Risk of bias assessment

The quality of the studies will be evaluated using the Risk of Bias in Systematic Review (ROBIS) tool [18,19]. This process involves three phases: (1) assessing relevance and consistency with the PICO principle, (2) evaluating data extraction and synthesis methods, and (3) assessing result interpretation and reporting processes. Based on these phases, the overall risk of bias will be classified as “low”, “unclear”, or “high”. Consensus will be reached between the two reviewers, and a third reviewer will be consulted in case of disagreements. The Risk of bias assessment process is illustrated in S3 File.

### 2.7. Assessment of methodological quality

Methodological quality will be independently evaluated by two reviewers using the Assessing the Methodological Quality of Systematic Reviews (AMSTAR 2) tool [20]. This instrument consists of 16 items, with seven key items (2, 4, 7, 9, 11, 13, and 15) being particularly important. AMSTAR 2 does not generate an overall score, instead, the methodological quality is classified as “high”, “moderate”, “low”, or “critically low” based on the ratings of key and non-key items. The Grading of Recommendations Assessment, Development, and Evaluation (GRADE) approach will be employed to assess the certainty of evidence for each primary outcome [21]. GRADE evaluates evidence quality across five dimensions: limitations, inconsistency, indirectness, imprecision, and publication bias, categorizing evidence as “high”, “moderate”, “low”, or “very low”. Small study effects will also be assessed using Egger’s test. Disagreements will be resolved through consensus or consultation with a third reviewer if necessary.

### 2.8. Data summary

Key characteristics of the included studies will be presented in tables, and the review results will be narrated. The umbrella review will be conducted using STATA 16.0 and R 4.2.1 software. The Corrected Covered Area (CCA) measure will be calculated to assess the degree of overlap in primary data. Binary variables will be synthesized using odds ratios (OR) or relative risks (RR) with 95% confidence intervals (95% CI), and continuous variables using standardized mean differences (SMD) and 95% CI. For mean difference (MD) effect sizes, data will be converted using Comprehensive Meta-Analysis 3.3.070. If aggregated results are unavailable, original data will be rigorously re-analyzed. Heterogeneity will be assessed using the *Q* test and **I*^*2*^* statistic. If *P* > 0.1 and **I*^*2*^* < 50%, a fixed-effects model will be used. If *P* ≤ 0.1 and **I*^*2*^* ≥ 50%, a random-effects model will be applied. Subgroup and sensitivity analyses will be conducted if the sources of heterogeneity remain unidentified. Publication bias will be assessed using funnel plots and the Egger test. For the qualitative systematic review, a narrative synthesis will be performed.

### 2.9. Analysis of subgroups or subsets

Subgroup analyses will be performed to explore the effects of aromatherapy attributes (e.g., population type, dosage, route of administration) and disease types on effect size. These analyses aim to identify predictors of improved outcomes.

### 2.10. Ethics and dissemination

The umbrella review does not require ethical approval because the information used is based solely on published articles. The findings will be published in peer-reviewed journals or presented at conferences.

## 3. Discussion

Globally, sleep is essential for health, yet an increasing number of individuals, including those with comorbidities, experience sleep disturbances, potentially delaying recovery and heightening medical risks. Improving patients’ sleep quality has therefore become an urgent issue. Aromatherapy, a widely recognized modality within CAT, has been extensively used to enhance sleep quality across various patient populations. With the rapid growth of clinical research publications on the effects of different types of aromatherapies on sleep quality, there has also been a corresponding increase in systematic reviews and meta-analyses. While these reviews offer insights into the effects of specific aromatherapy methods, such as inhalation and massage, a comprehensive evaluation of the evidence remains lacking.

To our knowledge, no umbrella review has yet provided a systematic assessment of the overall impact of aromatherapy on sleep quality. This umbrella review aims to fill that gap by offering a more comprehensive theoretical foundation for the clinical application of aromatherapy in improving sleep quality. It will evaluate the evidence in terms of methodological quality, reporting quality, and risk of bias. The findings from this review are expected to guide clinical decision-makers in implementing appropriate strategies to enhance sleep quality in patients. This is highly significant, as it aims to improve patient outcomes, optimize healthcare services, and reduce societal costs.

### 3.1. Strengths and limitations of this study

We will perform a systematic and comprehensive search across 10 databases, employing scientific and rigorous methods for data extraction and analysis. The quality of the included studies will be independently assessed by two reviewers using the AMSTAR 2 (Assessing the Methodological Quality of Systematic Reviews) tool. Additionally, the GRADE approach will be utilized to evaluate the certainty of evidence for each primary outcome.

This umbrella review aims to provide clinical decision-makers with more reliable and comprehensive evidence on the effects of aromatherapy on sleep quality in patients. However, including only Chinese and English databases may induce selection bias. Furthermore, overlaps among primary studies affecting data synthesis will be identified and noted in the tables.

### 3.2. Patient and public involvement

Patients and/or the public were not involved in the design, conduct, reporting, and dissemination plans of this research.

## Supporting information

S1 File PRISMA-P 2015 checklist. (DOCX)

S2 File Search strategies. (DOCX)

S3 File Risk of bias in systematic review (ROBIS) tool. (PDF)
