# Supplementary material for: Effects of aromatherapy on sleep quality in patients: Protocol for an umbrella review
Source: PLoS One. 2025 Aug 18;20(8):e0329928. doi: 10.1371/journal.pone.0329928 (PMC12360540; doi:10.1371/journal.pone.0329928)
Supplement: S2 File — (DOCX) [file pone.0329928.s002.docx]

**English database search strategies**

| Database | ID | Search strategy |
| --- | --- | --- |
| PubMed | #1 | (((((((((((((((aromatherapy[MeSH Terms]) OR (aroma*[Text Word])) OR (essential oil*[Text Word])) OR (fragrance[Text Word])) OR (oils, volatile[MeSH Terms])) OR (volatile oil*[Text Word])) OR (aroma therap*[Text Word])) OR (aromatic massage[Text Word])) OR (inhalational aroma[Text Word])) OR (inhalation aromatherapy[Text Word])) OR (massage aromatherapy[Text Word])) OR (incense therap*[Text Word])) OR (natural medicine[Text Word])) OR (Medicine, Traditional[MeSH Terms])) OR (phytotherapy[MeSH Terms])) OR (plants, medicinal[MeSH Terms]) |
|  | #2 | ((((((((((((sleep[MeSH Terms]) OR (sleep stages[MeSH Terms])) OR (Sleep quality[MeSH Terms])) OR (Sleep Deprivation[MeSH Terms])) OR (Sleep Wake Disorders[MeSH Terms])) OR (Sleep Initiation and Maintenance Disorders[MeSH Terms])) OR (Sleep Disorders, Circadian Rhythm[MeSH Terms])) OR (Sleep*[Text Word])) OR (Insomnia*[Text Word])) OR (somnolen*[Text Word])) OR (sleep apnea syndromes[MeSH Terms]) ) OR (hypersomnia[Text Word])) OR (parasomnia[Text Word]) |
|  | #3 | ((((((Systematic Review[Publication Type]) OR (Systematic review[Text Word])) OR (Systematic reviews[Text Word])) OR (Meta-analysis[Text Word])) OR (Meta-analyse[Text Word])) OR (Meta-analyses[Text Word])) OR (Network Meta-Analysis[MeSH Terms]) |
|  | #4 | #1 AND #2 AND #3 |
| Web Of Science | #1 | TS=(aromatherapy OR aroma* OR "essential oil*" OR fragrance OR "volatile oil*" OR "aroma therap*" OR "aromatic massage" OR "inhalational aroma" OR "inhalation aromatherapy" OR "massage aromatherapy" OR "incense therap*" OR "natural medicine" OR "Medicine, Traditional" OR phytotherapy OR "plants, medicinal") and Preprint Citation Index (Exclude – Database) |
|  | #2 | TS=(sleep* OR insomnia* OR hypersomnia OR parasomnia OR somnolen*) and Preprint Citation Index (Exclude – Database) |
|  | #3 | TS=("Systematic review*" OR "Meta-analys*") and Preprint Citation Index (Exclude – Database) |
|  | #4 | #1 AND #2 AND #3 |
| Embase  (Via Ovid) | 1 | exp aromatherapy/ |
|  | 2 | exp essential oil/ |
|  | 3 | exp fragrance/ |
|  | 4 | exp plant extract/ |
|  | 5 | exp traditional medicine/ |
|  | 6 | exp phytotherapy/ |
|  | 7 | exp medicinal plant/ |
|  | 8 | (aroma* or "volatile oil*" or "oils, volatile" or "essential oil*" or "incense therap*" or fragrance or "medicinal plant" or "traditional medicine" or "plant extract" or phytotherapy or "natural medicine").ab,ti. |
|  | 9 | 1 or 2 or 3 or 4 or 5 or 6 or 7 or 8 |
|  | 10 | exp sleep/ |
|  | 11 | exp sleep deprivation/ |
|  | 12 | exp sleep apnea syndromes/ |
|  | 13 | exp sleep disorder/ |
|  | 14 | exp circadian rhythm sleep disorder/ |
|  | 15 | exp sleep quality/ |
|  | 16 | exp sleep stage/ |
|  | 17 | exp insomnia/ |
|  | 18 | exp parasomnia/ |
|  | 19 | exp hypersomnia/ |
|  | 20 | exp somnolence/ |
|  | 21 | (sleep* or Insomnia* or somnolen* or hypersomnia or parasomnia).ab,ti. |
|  | 22 | 10 or 11 or 12 or 13 or 14 or 15 or 16 or 17 or 18 or 19 or 20 or 21 |
|  | 23 | "systematic review"/ |
|  | 24 | meta analysis/ |
|  | 25 | ("systematic review" or "meta analys*" or meta-analys*).ti. |
|  | 26 | 23 or 24 or 25 |
|  | 27 | 9 and 22 and 26 |
| JBI (Via Ovid) | 1 | exp aromatherapy/ |
|  | 2 | exp Oils, Volatile / |
|  | 3 | exp Odorants / |
|  | 4 | exp plant extract/ |
|  | 5 | exp Medicine, Tradition/ |
|  | 6 | exp phytotherapy/ |
|  | 7 | exp Plants, Medicinal / |
|  | 8 | (aroma* or "volatile oil*" or "oils, volatile" or "essential oil*" or "incense therap*" or fragrance or "medicinal plant" or "traditional medicine" or "plant extract" or phytotherapy or "natural medicine").ab,ti. |
|  | 9 | 1 or 2 or 3 or 4 or 5 or 6 or 7 or 8 |
|  | 10 | exp sleep/ |
|  | 11 | exp sleep deprivation/ |
|  | 12 | exp sleep apnea syndromes/ |
|  | 13 | exp Sleep Wake Disorders / |
|  | 14 | exp Sleep Disorders, Circadian Rhythm / |
|  | 15 | exp sleep quality/ |
|  | 16 | exp sleep stage/ |
|  | 17 | Exp "Sleep Initiation and Maintenance Disorders"/ |
|  | 18 | exp parasomnia/ |
|  | 19 | exp Disorders of Excessive Somnolence / |
|  | 20 | exp Sleepiness / |
|  | 21 | (sleep* or Insomnia* or somnolen* or hypersomnia or parasomnia).ab,ti. |
|  | 22 | 10 or 11 or 12 or 13 or 14 or 15 or 16 or 17 or 18 or 19 or 20 or 21 |
|  | 23 | "systematic review"/ |
|  | 24 | meta analysis/ |
|  | 25 | ("systematic review" or "meta analys*" or meta-analys*).ab,ti. |
|  | 26 | 23 or 24 or 25 |
|  | 27 | 9 and 22 and 26 |
| Cochrane Library | #1 | MeSH descriptor: [Aromatherapy] explode all trees |
|  | #2 | MeSH descriptor: [Oils, Volatile] explode all trees |
|  | #3 | MeSH descriptor: [Medicine, Traditional] explode all trees |
|  | #4 | MeSH descriptor: [Phytotherapy] explode all trees |
|  | #5 | MeSH descriptor: [Plants, Medicinal] explode all trees |
|  | #6 | (aroma* OR 'essential oil*' OR fragrance OR 'volatile oil*' OR 'aroma therap*' OR 'aromatic massage' OR 'inhalational aroma' OR 'inhalation aromatherapy' OR 'massage aromatherapy' OR 'incense therap*' OR 'natural medicine'):ti,ab,kw |
|  | #7 | MeSH descriptor: [Sleep] explode all trees |
|  | #8 | (sleep*):ti,ab,kw OR (Insomnia*):ti,ab,kw OR (somnolen*):ti,ab,kw OR (hypersomnia):ti,ab,kw OR (parasomnia):ti,ab,kw |
|  | #9 | MeSH descriptor: [Sleep Deprivation] explode all trees |
|  | #10 | MeSH descriptor: [Sleep Stages] explode all trees |
|  | #11 | MeSH descriptor: [Sleep Quality] explode all trees |
|  | #12 | MeSH descriptor: [Sleep Wake Disorders] explode all trees |
|  | #13 | MeSH descriptor: [Sleep Apnea Syndromes] explode all trees |
|  | #14 | MeSH descriptor: [Sleep Initiation and Maintenance Disorders] explode all trees |
|  | #15 | MeSH descriptor: [Sleep Disorders, Circadian Rhythm] explode all trees |
|  | #16 | (Meta-Analysis):pt OR (systematic review):pt OR ('Systematic review*'):ti,ab,kw OR (Meta-analys*):ti,ab,kw |
|  | #17 | MeSH descriptor: [Systematic Review] explode all trees |
|  | #18 | MeSH descriptor: [Meta-Analysis] explode all trees |
|  | #19 | #1 OR #2 OR #3 OR #4 OR #5 OR #6 |
|  | #20 | #7 OR #8 OR #9 OR #10 OR #11 OR #12 OR #13 OR #14 OR #15 |
|  | #21 | #16 OR #17 OR #18 |
|  | #22 | #19 AND #20 #21 |
| CINAHL Plus EBSCOhost | S1 | (MH "Aromatherapy") |
|  | S2 | (MH "Medicine, Traditional") |
|  | S3 | (MH "Medicine, Herbal") |
|  | S4 | (MH "Plants, Medicinal") |
|  | S5 | SU ("aroma*" OR "essential oil*" OR "fragrance" OR "oils, volatile"  OR "volatile oil*" OR "aromatherap*" OR "aromatic massage"  OR "inhalational aroma" OR "inhalation aromatherapy" OR "massage aromatherapy" OR "incense therap*" OR "naturalmedicine" OR "phytotherapy") |
|  | S6 | S1 OR S2 OR S3 OR S4 OR S5 |
|  | S7 | (MH "Sleep Disorders, Circadian Rhythm") OR (MH "Parasomnias") |
|  | S8 | (MH "Sleep") OR (MH "Sleep Disorders") OR (MH "Sleep Quality") OR (MH "Sleep Deprivation") OR (MH "Sleep Stages") OR (MH "Sleep Apnea Syndromes") |
|  | S9 | (MH "Insomnia") |
|  | S10 | SU (sleep* OR Insomnia* OR somnolen* OR hypersomnia OR parasomnia) |
|  | S11 | S7 OR S8 OR S9 OR S10 |
|  | S12 | (MH "systematic review") OR (MH "meta analysis") |
|  | S13 | SU ("systematic review*" OR "meta analys*" OR meta-analys*) |
|  | S14 | S12 OR S13 |
|  | S15 | S6 AND S11 AND S14 |

**Chinese database search strategy**

**Chinese search strategy:** (芳香疗法 OR 芳疗 OR 香疗 OR 精油 OR 熏香 OR 精油按摩 OR 吸入性芳香疗法 OR 芳香吸嗅疗法 OR 芳香类物质治疗 OR 芳香植物疗愈 OR 芳香按摩疗法) AND (睡眠 OR 睡眠质量 OR 失眠) AND (系统评价 OR Meta分析)

**Chinese search strategy in Pinyin:** (fangxiangliaofa OR fangliao OR xiangliao OR jingyou OR xunxiang OR jingyouanmo OR xiruxingfangxiangliaofa OR fangxiangxixiuliaofa OR fangxiangleiwuzhizhiliao OR fangxiangzhiwuliaoyu OR fangxianganmoliaofa) AND (shuimian OR shuimianzhiliang OR shimian) AND (xitongpingjia OR Metafenxi)

**Chinese search strategy in translation:** (aromatherapy OR fragrance therapy OR aroma therapy OR essential oil OR incense OR essential oil massage OR inhalation aromatherapy OR aromatic inhalation therapy OR aromatic substance therapy OR aromatic plant therapy OR aromatherapy massage) AND (sleep OR sleep quality OR Insomnia) AND (systematic review OR meta analysis)
